# Effects of long non-coding RNA Gm14461 on pain transmission in trigeminal neuralgia

**DOI:** 10.1186/s12950-019-0231-1

**Published:** 2020-01-03

**Authors:** Mu Xu, Yi Yan, Mengye Zhu, Zhijian Wang, Xuexue Zhang, Daying Zhang

**Affiliations:** Department of Pain Medicine, The first affiliated hospital of NanChang university, 17 Yongwai Zhengjie, Donghu District, Nanchang, 330006 Jiangxi China

**Keywords:** Trigeminal neuralgia, Gm14461, Inflammation, CGRP, P2X3 receptor, P2X7 receptor

## Abstract

**Background:**

This study aims to investigate the role of long non-coding RNA Gm14461 in regulating pain transmission in trigeminal neuralgia (TN). The mouse TN model was produced by chronic constriction injury of the infraorbital nerve (CCI-ION). The values of mechanical withdrawal threshold (MWT) were measured to assess the nociception of mice at different times after CCI-ION surgery (0, 1, 3, 5, 7, 9, 11, 13, 15 d). The primary mouse trigeminal ganglion neurons (TGNs) were isolated from C57BL/6 J mice and treated with TNF-α to mimic a TN cellular model. The expression of Gm14461, TNF-α, IL-1β, and IL-6 was examined using qRT-PCR. The protein levels of CGRP and P2X3/7 receptor were measured using western blot.

**Results:**

Gm14461 expression was increased in trigeminal ganglia (TGs) of TN mice on the operation side. Furthermore, Gm14461 knockdown in TGs increased, whereas Gm14461 overexpression decreased MWT in TN mice. Moreover, Gm14461 knockdown downregulated, whereas Gm14461 overexpression upregulated mRNA levels of TNF-α, IL-1β, and IL-6 and protein levels of CGRP and P2X3/7 receptor in TGs from TN mice. In vitro assay showed that Gm14461 was upregulated by TNF-α, IL-1β, and IL-6. Additionally, Gm14461 knockdown decreased protein levels of CGRP and P2X3/7 receptor in TNF-α-treated TGNs, whereas Gm14461 overexpression exerted the opposite effect.

**Conclusion:**

Gm14461 promoted pain transmission (reduced MWT value) in a CCI-ION-induced mouse TN model. The underlying mechanisms might involve the regulation of pro-inflammatory cytokines, CGRP and P2X3/7 receptor.

## Introduction

Trigeminal neuralgia (TN) is a common type of neuropathic pain and characterized by severe, episodic pain in the trigeminal nerve distribution [1]. Currently, the main treatments for TN include medical therapy, microvascular decompression, percutaneous radiofrequency rhizotomy, and stereotactic radiosurgery [2]. However, treatment effects are not completely satisfying. Hence, it is of great importance to search for new molecular targets for the prevention and treatment of TN.

Long non-coding RNAs (lncRNAs) are commonly defined as non-protein-coding RNA transcripts with lengths exceeding 200 nucleotides and involved in the regulation of gene transcription, translation, epigenetic modification and so on [3]. Emerging evidence indicates that lncRNAs are deregulated and play important roles in the development of neuropathic pain [4, 5]. Wang et al. [6] suggested that the expression levels of lncRNA uc.48+ and purinoceptor P2X3 receptor in the diabetic rat dorsal root ganglia (DRG) were increased, and that the mechanical withdrawal threshold (MWT) and thermal withdrawal latency (TWL) values in rats with diabetic neuropathic pain treated with lncRNA uc.48+ siRNA were increased [6]. The P2X3 receptor, belongs to the P2X receptor family (P2X1–7), plays a crucial role in pain transmission at peripheral sensory neurons [7]. Recently, Xiong et al. [8] also found that lncRNA uc.48 + siRNA significantly reversed the reduced MWT in the chronic constriction injury (CCI) of the infraorbital nerve (ION) (CCI-ION)-induced TN rats. Evidence also suggested that NONRATT021972 siRNA treatment in type 2 diabetic rats increased the MWT, TWL and the sensory nerve conduction velocity (SNCV) of rat tail nerves [9, 10]. The afore-mentioned findings suggest the potential of lncRNAs as therapeutic targets for neuropathic pain.

A previous study showed that lncRNA Musmusculus predicted gene 14,461 (Gm14461) was highly expressed in injured DRG 6 days after spinal nerve ligation in mice with neuropathic pain, suggesting that Gm14461 may be involved in regulating neuropathic pain [11]. Diverse causes of neuropathic pain are associated with excessive inflammation [12, 13]. Cytokines tumor necrosis factor (TNF)-α, interleukin (IL)-1β, and IL-6 play a very important role in the pathogenesis of TN by regulating the immune response within the peripheral endings of trigeminal ganglion neurons (TGNs) [14]. In addition, calcitonin gene-related peptide (CGRP) [15], purinoceptors P2X3 receptor [7] and P2X7 receptor [9] play a crucial role in the trigeminal nociceptive process. In this study, we sought to elucidate whether Gm14461 was also involved in TN pain transmission. Furthermore, we investigated the role of Gm14461 in regulating inflammatory response and expression of CGRP and P2X3/7 receptor in TN.

## Materials and methods

### Animals

C57BL/6 J mice (weight 13–17 g, 4 to 6 weeks old) were raised under a specific pathogen-free environment (temperature, 22 ± 0.5 °C; humidity, 45–55%) in a 12 h light/12 h dark cycle throughout the experimental period. Animals were habituated for 1 week before experiments. This study was approved by the Ethics Committee of The first affiliated hospital of NanChang university and followed the ethical guidelines of the International Association for the Study of Pain (IASP) for pain research in animals.

### Animal experimental design

C57BL/6 J mice were randomly divided into six groups (*n* = 8/group): Sham, TN, TN + Scramble siRNA, TN + si-Gm14461, TN + Vector, and TN + Gm14461 group. The mice in the TN + Scramble siRNA, TN + si-Gm14461, TN + Vector, and TN + Gm14461 group received a local injection of the scramble siRNA, Gm14461 siRNA (Gm14461 knockdown plasmids), pcDNA3.1 empty plasmids and pcDNA3.1-Gm14461 (Gm14461 overexpression plasmids) respectively via infraorbital foramen to trigeminal ganglia (TGs) continuously for 3 days [8].

### Establishment of a mouse model of TN

The mouse TN model was produced by CCI-ION via an intraoral approach as described previously [16]. Briefly, the animal was anesthetized with 4% sodium pentobarbital (50 mg/kg) by intraperitoneal injection. Then the head was fixed and the mouth kept open during the operation. A 1-cm incision was made along the left gingivobuccal margin in the buccal mucosa, beginning immediately next to the first molar. The left infraorbital nerve was freed and loosely tied with 2 to 4 chromic gut (4–0) ligatures 1.5–2 mm apart, after which the incision was sutured. Compression criteria: the ligature significantly reduces the diameter of the nerve, slowing but not blocking circulation through the superficial blood vessels. The animals in the sham operation group received only nerve exposure but no ligation. The contralateral side of all mice remained intact. All surgical procedures were performed under aseptic conditions.

### MWT measurement

The MWT was measured to assess the nociception of mice at different times after CCI-ION surgery (0, 1, 3, 5, 7, 9, 11, 13, 15 d). The measurement time was constant from 10:00 to 14:00 daily. Briefly, the mice were placed in a separate clear plastic chamber (22 × 12 × 22 cm) on a stainless-steel mesh floor for acclimation for at least one hour. Subsequently, a series of calibrated *von* Frey filaments (Stoelting, Wood Dale, IL, USA) was lightly applied to the skin within the infraorbital territory, near the center of the vibrissal pad on hairy skin surrounding the mystacial vibrissae. Stimulation was performed in increasing intensity until a withdrawal response occurred or the force reached 2.0 g (the cut-off value). Each filament was tested 5 times at 5-s intervals. Rapid retreat, dodge, turn, attack, and scratch actions were defined as positive responses. The MWT was defined as the lowest force in grams that produced at least three positive responses in 5 consecutive applications.

### Isolation and culture of mouse TGNs

TGNs were isolated from 4 to 6 week-old C57BL/6 mice. Briefly, the mice were sacrificed by cervical dislocation and the head was disinfected with 75% alcohol. The scalpel was used to quickly cut the skin and skull, and the brain tissue was opened to expose the TGs. The TGs were removed with a forceps and a scalpel under a dissecting microscope, and the fibrous tissue was cut off with fine forceps and scissors in pre-cooled PBS (pH 7.4). Afterward, the TGs were digested in 0.25% collagenase for 25 min and 0.5% trypsin for 15 min, and cultured in Dulbecco’s Modified Eagle Medium (DMEM) medium containing 10% fetal bovine serum (FBS). Following disperse into a single-cell suspension with gentle pipetting, the TGNs were cultured at 0.1% polylysine-coated slides in humidified air at 37 °C with 5% CO_2_. Two hours later, the cells were cultured in DMEM medium supplemented with2.5% FBS, 2% B27, 0.05 mg/mL penicillin, 0.05 mg/mL streptomycin, and 0.1% mg/mL neomycin.

### Plasmid construction, cell transfection and treatment

To overexpress Gm14461, the full-length Gm14461 were cloned into the pcDNA 3.1 plasmid (Invitrogen; Thermo Fisher Scientific), generating pcDNA3.1-Gm14461 plasmids. An empty pcDNA3.1 vector was used as the control. To knock down Gm14461, small interfering RNA (siRNA) targeting Gm14461 (si-Gm14461) was purchased from Thermo Fisher Scientific. A scramble siRNA was used as negative control. The primary TGNs were transfected with these constructs using Lipofectamine® 3000 (Invitrogen; Thermo Fisher Scientific), according to the manufacturer’s instructions.

The primary TGNs were treated with TNF-α (10 ng/mL), IL-1β (25 ng/mL) and IL-6 (25 ng/mL) for 24 h. Then Gm14461 expression in TGNs was examined. In another experiment, the primary TGNs were transfected with scramble siRNA, si-Gm14461, empty vector, or pcDNA3.1-Gm14461 in the stimulation of TNF-α (10 ng/mL) for 24 h. Western blot analysis of protein levels of CGRP, P2X3 receptor, and P2X7 receptor in TGNs.

### Quantitative real-time PCR (qRT-PCR)

Total RNA was extracted from mouse ipsilateral TGs on the operation side and primary TNGs using TRIzol (Invitrogen) and reverse transcribed using the ThermoScript reverse transcriptase (Invitrogen) according to the manufacturer’s protocol. The cDNA templates were amplified through qRT-PCR using Advanced Universal SYBR Green Supermix (Bio-Rad Laboratories, Hercules, CA, USA) in a CFX96 real-time PCR system (Bio-Rad Laboratories). The relative expression of candidate genes was calculated by the 2^-ΔΔCt^ method and normalized to the internal control β-actin.

### Western blot

Total protein was extracted from mouse ipsilateral TGs on the operation side and primary TGNs in lysis buffer. Mouse ipsilateral TGs on the operation side were isolated, rinsed with ice-cold PBS, and then homogenized by mechanical disruption (grinding) in lysis buffer. Following incubation on ice for 50 min, the homogenate was then pelleted at 12,000 rpm for 5 min and the supernatant was collected. The protein concentration was quantified with the bicinchoninic acid kit. Samples containing equal amounts of protein (20 μg) were separated using 10% SDS-PAGE gels and electroblotted onto PVDF membranes (Millipore, USA). The membrane was then blocked with 5% non-fat dry milk and incubated with the primary antibodies against the CGRP, P2X3, and P2X7 (1:1000; all purchased from Santa Cruz Biotechnology). The membrane was then washed in TBST and incubated with horseradish peroxidase (HRP)-conjugated IgG (Santa Cruz Biotechnology). The bands were visualized using an enhanced chemiluminescence kit (ECL kit, Pierce Biotechnology, IL). The band densities were normalized against a β-actin internal control. The quantification was performed using Image J software.

### Statistical analysis

All statistical analyses were performed using SPSS 16.0 (SPSS, Inc., Chicago, IL, USA). The data are presented as the mean ± standard deviation (SD). The unpaired Student’s *t*-test and one-way analysis of variance (ANOVA) were used to analyze differences between two groups and multiple groups, respectively. *p* < 0.05 was considered to indicate a statistically significant difference.

## Results

### Gm14461 expression was increased in TN mice

**Fig. 1:**
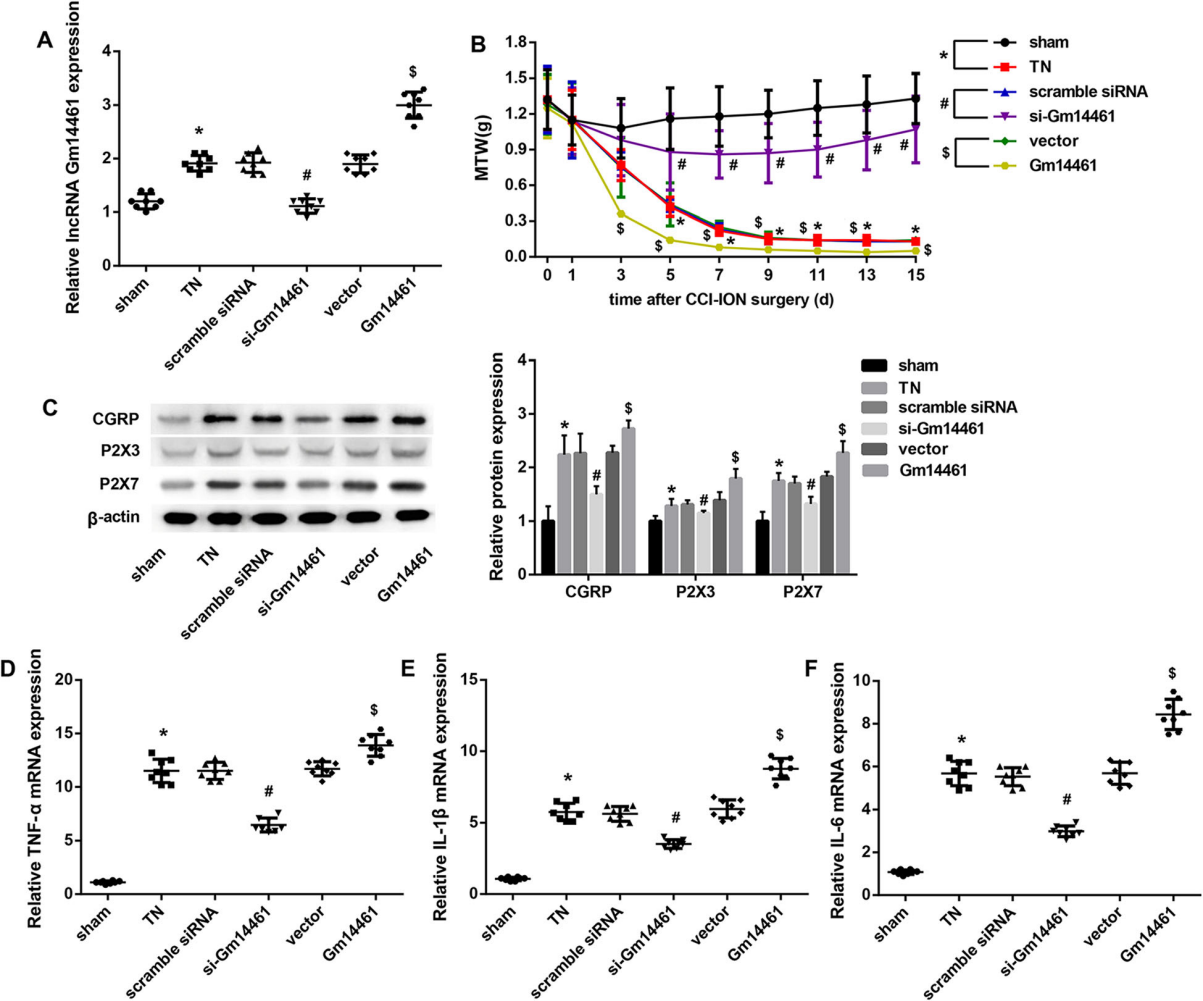
Effect of Gm14461 expression on MWT and expression of Gm14461, CGRP, P2X3 receptor, P2X7 receptor, TNF-α, IL-1β, and IL-6 in TN mice. C57BL/6 J mice were randomly divided into six groups (*n* = 8/group): Sham, TN, TN + Scramble siRNA, TN + si-Gm14461, TN + Vector, and TN + Gm14461 group. **a** Gm14461 expression in mouse TGs was detected by qRT-PCR. **b** The MWT was measured to assess the nociception of mice at different times after CCI-ION surgery (0, 1, 3, 5, 7, 9, 11, 13, 15 d). ^*^P < 0.05: Sham vs. TN; ^#^*P* < 0.05 Scramble siRNA vs. si-Gm14461; ^$^*P* < 0.05 Vector vs. Gm14461. **c** The protein levels of CGRP, P2X3 receptor, and P2X7 receptor were examined using western blot. **d-f** The mRNA levels of TNF-α (**d**), IL-1β (e), and IL-6 (f) in mouse TGs were detected by qRT-PCR. Data are presented as mean ± SD. ^*^P < 0.05 vs. Sham; ^#^*P* < 0.05 vs. Scramble siRNA; ^$^*P* < 0.05 vs. Vector

The Gm14461 expression in TGs from mice in the TN group was significantly higher than that in the sham group. Furthermore, Gm14461 siRNA diminished the upregulated Gm14461 expression in TGs from TN mice on the operation side. We also found that Gm14461 overexpression further increased Gm14461 expression in TGs from TN mice on the operation side **(**Fig. 1a**)**.

### Gm14461 knockdown increased, whereas Gm14461 overexpression decreased MWT in TN mice

The MWT was measured to assess the nociception of mice at different times after CCI-ION surgery (0, 1, 3, 5, 7, 9, 11, 13, 15 d). Compared with the Sham-operated mice, the experimental TN mice had a significantly decreased MWT. Furthermore, compared with the scramble siRNA group, the mice in the si-Gm14461 showed a notable increase in MWT. In contrast, the MWT in the Gm14461 group was markedly decreased when compared with the Vector group **(**Fig. 1b**)**.

### Gm14461 knockdown downregulated, whereas Gm14461 overexpression upregulated expression of CGRP and P2X3/7 receptor in TN mice

Compared with the Sham-operated mice, the protein levels of CGRP and P2X3/7 receptor in TGs from TN mice on the operation side were notably increased, which were decreased by Gm14461 knockdown. On the contrary, Gm14461 overexpression significantly increased protein levels of CGRP, P2X3 receptor, and P2X7 receptor in TGs from TN mice **(**Fig. 1c**)**.

### Gm14461 knockdown inhibited, whereas Gm14461 overexpression induced mRNA levels of inflammatory cytokines in TN mice

Compared with the Sham-operated mice, the mRNA levels of inflammatory cytokines including TNF-α **(**Fig. 1d**)**, IL-1β **(**Fig. 1e**)**, and IL-6 **(**Fig. 1f**)** in TGs from TN mice were notably increased, which were decreased by Gm14461 knockdown. On the contrary, Gm14461 overexpression significantly increased mRNA levels of TNF-α, IL-1β, and IL-6 in TGs from TN mice **(**Fig. 1d-f**)**.

### Gm14461 knockdown downregulated, whereas Gm14461 overexpression upregulated expression of CGRP and P2X3/7 receptor in TNF-α-treated mouse TGNs

**Fig. 2:**
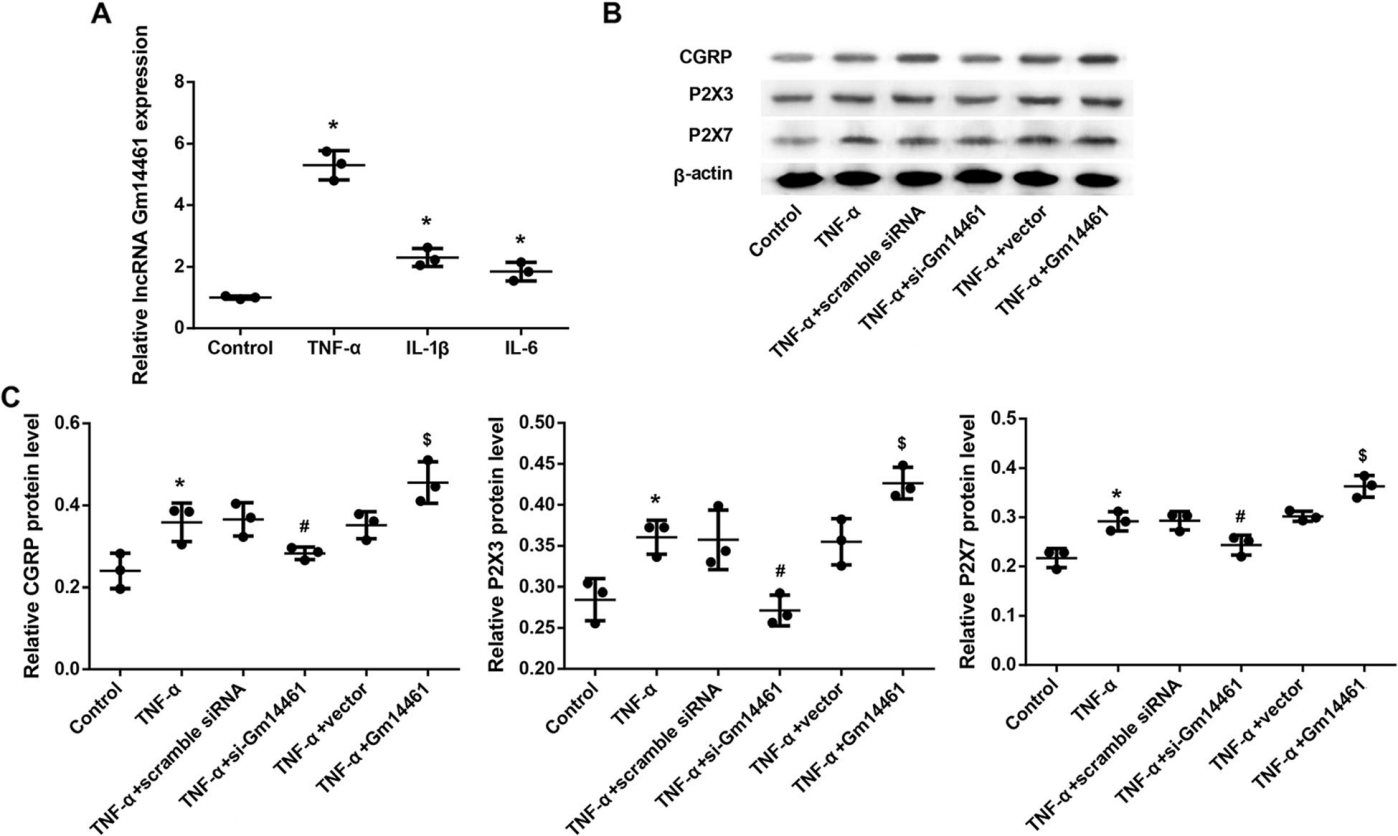
Effect of Gm14461 expression on the expression of CGRP, P2X3 receptor, and P2X7 receptor in primary TGNs. **a** The primary mouse TGNs were isolated from C57BL/6 J mice and treated with TNF-α (10 ng/mL), IL-1β (25 ng/mL) and IL-6 (25 ng/mL) for 24 h. Gm14461 expression in TGNs was examined by qRT-PCR. ^*^P < 0.05 vs. Control. **b** Western blot analysis of protein levels of CGRP, P2X3 receptor, and P2X7 receptor in primary TGNs transfected with scramble siRNA, si-Gm14461, empty vector, and pcDNA3.1-Gm14461 in the stimulation of TNF-α (10 ng/mL) for 24 h. **c** The qualifications of western blots in (**b**). Data are presented as mean ± SD. *N* = 3. ^*^P < 0.05 vs. Control; ^#^*P* < 0.05 vs. TNF-α + Scramble siRNA;^$^*P* < 0.05 vs. TNF-α + Vector

The pro-inflammatory cytokines TNF-α, IL-1β, and IL-6 significantly increased Gm14461 expression in primary mouse TGNs **(**Fig. 2a**)**. Furthermore, treatment with TNF-α also notably upregulated protein levels of CGRP and P2X3/7 receptor **(**Fig. 2b-c**)**. Gm14461 knockdown significantly diminished the TNF-α-induced levels of CGRP and P2X3/7 receptor **(**Fig. 2b-c**)**, whereas Gm14461 overexpression further increased the protein levels of CGRP and P2X3/7 receptor in TNF-α-treated TGNs **(**Fig. 2b-c**)**.

## Discussion

TN seriously affects the quality of life of the patients and is difficult to cure. Currently, the most widely accepted explanation for the pathogenesis of primary TN is the microvascular compression theory. At present, CCI-ION has been generally used to establish an animal model of TN [17]. The MWT value in our CCI-ION-induced TN mice was decreased after the CCI-ION operation and deteriorated for the following 15 days, which was consistent with previous studies [8].

It is currently believed that the molecular mechanisms underlying the pathogenesis of TN involve changes in a variety of pain-related neuropeptides and receptors. CGRP is expressed by calcitonin Cal/CGRP gene and is a biologically active polypeptide with pain-promoting effect. The neurotransmitter CGRP plays a crucial role in trigeminal nociceptive processing [15]. It is well known that activation of the trigeminal nerve system induces the release of CGRP [15] and blocking of CGRP receptor may be one of the ways to treat TN [18]. The P2X3 receptor and P2X7 receptor play a crucial role in facilitating pain transmission in TN [7, 19, 20]. P2X3 receptor and P2X7 receptor belong to the family of purinoceptors for ATP and function as ligand-gated ion channels and may transduce ATP-evoked nociceptor activation [7, 8]. Here, we observed an increase in expression of CGRP, P2X3 receptor and P2X7 receptor in TGs from TN model mice, which was consistent with previous studies [8, 19].

Pro-inflammatory cytokines TNF-α, IL-1β, and IL-6 play a very important role in the pathogenesis of TN by regulating the immune response within the peripheral endings of TGNs [14]. Some cytokines (e.g., TNF-α and IL-1β) have also been demonstrated to sensitize nociceptive neurons [14]. Our results here showed that mRNA levels of TNF-α, IL-1β, and IL-6 were significantly upregulated in TGs from TN mice when compared with the sham-operated mice, indicating that inflammatory mediators may also be pathogenic factors of TN. In vitro culture of TGNs in the presence of cytokines such as TNF-α can mimic neurogenic pain under inflammatory condition [20]. It has been reported that infraorbital foramen injection of TNF-α and IL-1β successfully produced a rat TN model with TN-like symptoms and pathological changes, which further confirmed that inflammatory factors are involved in the pathogenesis of TN [21]. In this study, we treated TGNs with TNF-α to mimic a TN cellular model. Our results demonstrated that similar to the increased Gm14461 in TN mouse model, Gm14461 expression was also increased in TGNs treated with TNF-α, IL-1β, and IL-6. Furthermore, the protein levels of CGRP and P2X3/7 receptor were also upregulated in TNF-α-treated TGNs.

Emerging evidence indicates that lncRNAs are deregulated and play important roles in the development of neuropathic pain [4, 5]. For example, Li et al. [22] reported that lncRNA MRAK009713 expression was markedly increased in CCI rats, and siRNA against MRAK009713 significantly inhibited the nociceptive P2X3 expression and thus reduced both mechanical and thermal hyperalgesia in the CCI rats. Gm14461 was highly expressed in injured DRG 6 days following peripheral nerve injury in mice with neuropathic pain, suggesting the potential involvement of Gm14461 in regulating neuropathic pain [11]. Here, we found that lncRNAGm14461 expression was upregulated in TGs of ION-CCI-induced TN model mice when compared with the sham-operated mice. TN could occur due to the primary abnormality of increased hyperexcitation in the TG and trigeminal root. Thus our results indicated that Gm14461 may be associated with the pain transmission of TN. To address the functional role of Gm14461 in TN, this study knocked down and overexpressed Gm14461 in ION-CCI-induced TN model mice and in TNF-α-treated TGNs, and then examined levels of pro-inflammatory cytokines, CGRP, and P2X3/7 receptor.

Our findings showed that Gm14461 knockdown increased, whereas Gm14461 overexpression decreased MWT in TGs from TN mice. The reduced mechanical withdrawal threshold was associated with increased pain transmission. Thus, our results suggested that Gm14461 might have a regulatory role in mouse mechanical hyperalgesia, and could be a potential target for TN treatment. Furthermore, Gm14461 was increased by TNF-α, IL-1β, and IL-6. We also found that Gm14461 knockdown decreased, whereas Gm14461 overexpression increased mRNA levels of TNF-α, IL-1β, and IL-6 in TGs from TN mice. Moreover, Gm14461 knockdown downregulated, whereas Gm14461 overexpression upregulated, the induced protein levels of CGRP and P2X3/7 receptor in ION-CCI-induced TN model mice and in TNF-α-treated TGNs. In light of the important role of inflammatory cytokines, CGRP and P2X3/7 receptor in trigeminal nociceptive processing, we may suggest that, Gm14461 might promote pain transmission (reduce MWT value) via promoting inflammatory response and inducing expression of CGRP and P2X3/7 receptor in TGs under TN situation. Our in vivo results showed that in the si-Gm14461 group there was a dramatic decrease in CGRP relative protein expression but a minimal decrease in P2X3 protein expression, indicating that CGRP might act as main driver of pain in the TN model. Accordingly, we hypothesized that Gm14461 might promote pain transmission, mostly likely, through inducing CGRP expression.

## Conclusion

In conclusion, Gm14461 overexpression promoted pain transmission (reduced MWT value), whereas Gm14461 knockdown inhibited pain transmission (elevated MWT value) in a CCI-ION-induced mouse TN model. The underlying mechanisms might involve the regulation of expression of pro-inflammatory cytokines, CGRP and P2X3/7 receptor.

## Data Availability

The datasets used and/or analyzed during the current study are available from the corresponding author on reasonable request.
